# Impact of Learning to Read in a Mixed Approach on Neural Tuning to Words in Beginning Readers

**DOI:** 10.3389/fpsyg.2019.03043

**Published:** 2020-01-23

**Authors:** Alice van de Walle de Ghelcke, Bruno Rossion, Christine Schiltz, Aliette Lochy

**Affiliations:** ^1^Psychological Sciences Research Institute and Institute of Neuroscience, Université catholique de Louvain, Louvain-la-Neuve, Belgium; ^2^CNRS-CRAN, Université de Lorraine, Nancy, France; ^3^Service de Neurologie, CHRU-Nancy, Université de Lorraine, Nancy, France; ^4^Department of Behavioral and Cognitive Sciences, Faculty of Humanities, Social and Educational Sciences, Institute of Cognitive Science and Assessment, Université du Luxembourg, Esch-sur-Alzette, Luxembourg

**Keywords:** FPVS-EEG, development, teaching methods, reading, poor readers

## Abstract

The impact of learning to read in a mixed approach using both the global and phonics teaching methods on the emergence of left hemisphere neural specialization for word recognition is yet unknown in children. Taking advantage of a natural school context with such a mixed approach, we tested 42 first graders behaviorally and with Fast Periodic Visual Stimulation using electroencephalographic recordings (FPVS-EEG) to measure selective neural responses to letter strings. Letter strings were inserted periodically (1/5) in pseudofonts in 40 s sequences displayed at 6 Hz and were either words globally taught at school, that could therefore be processed by visual whole-word form recognition (global method), or control words/pseudowords eliciting grapheme-phoneme (GP) mappings (phonics method). Results show that selective responses (F/5, 1.2 Hz) were left lateralized for control stimuli that triggered GP mappings but bilateral for globally taught words. It implies that neural mechanisms recruited during visual word processing are influenced by the nature of the mapping between written and spoken word forms. GP mappings induce left hemisphere discrimination responses, and visual recognition of whole-word forms induce bilateral responses, probably because the right hemisphere is relatively more involved in holistic visual object recognition. Splitting the group as a function of the mastery of GP mappings into “good” and “poor” readers strongly suggests that good readers actually processed all stimuli (including global words) predominantly with their left hemisphere, while poor readers showed bilateral responses for global words. These results show that in a mixed approach of teaching to read, global method instruction may induce neural processes that differ from those specialized for reading in the left hemisphere. Furthermore, given their difficulties in automatizing GP mappings, poor readers are especially prone to rely on this alternative visual strategy. A preprint of this paper has been released on Biorxiv (van de Walle de Ghelcke et al., 2018).

## Introduction

Reading is an essential prerequisite for the acquisition of knowledge across all school disciplines. It is also a complex skill, acquired only with formal instruction. Yet, little is known about how different teaching methods influence the development of neural circuits for reading. This lack of knowledge is surprising given its critical relevance for pedagogical and clinical purposes. In many primary schools, first grade teachers rely on a so-called “mixed” approach, using at least two different methods in parallel for teaching to read. On the one hand, the “global” method requires children to visually memorize words globally. On the other hand, the phonics method teaches them letters-speech sounds mappings (grapheme-phoneme mappings, GP hereafter). In a natural school context using such a mixed approach with the “global” and the “phonics” methods, the present study assessed the potential cortical impact of the different cognitive processes induced by these methods in first grade children. Specifically, we compared neurophysiological responses to words that have been taught globally to control letter strings (words/pseudowords) that rely on GP mappings. In order to disentangle the potential role of familiarity by itself (intrinsically linked with the global method which involves an item-by-item learning), we then examined if responses to global words vary according to the mastery of GP mappings in two groups of children similarly exposed to global words.

Adults’ expert reading is characterized by highly automated recognition of written words. This automaticity allows accurate, effortless and fast (200 ms per word; Rayner, 1998; Rayner et al., 2012) access to words representation integrating their orthographic, phonological and semantic properties. It is widely acknowledged that a subregion of the left ventral occipito-temporal cortex (L-VOTC) termed the “Visual Word Form Area” (“VWFA,” Cohen et al., 2000) is crucial for the fast recognition of written words (see also Lochy et al., 2018 for electrophysiological intracerebral evidence). Through putative connections with phonological and lexico-semantic systems, the VWFA is thought to trigger access to words’ phonological and semantic properties (Jobard et al., 2003; McCandliss et al., 2003).

Before becoming fluent readers, children undergo laborious formal instruction during the first 2 years of primary school. Developmental studies have shown that the specialization of the posterior left hemisphere for reading is driven by children’s early reading experience and reading acquisition (Maurer et al., 2006; Schlaggar and McCandliss, 2007; Brem et al., 2010; Eberhard-Moscicka et al., 2015; Lochy et al., 2016; Dehaene-Lambertz et al., 2018). In line with the Phonological Mapping Hypothesis (Maurer and McCandliss, 2007), this left hemispheric specialization is thought to emerge during the learning of GP mappings, inducing progressive connections between posterior visual regions (letters representations) and anterior language-related regions (speech sounds representations).

Developmental models suggest that expert reading is built from the automatization of analytical processes performed on written words (e.g., orthographic, visuo-attentional, phonological) (e.g., Frith et al., 1985; Perfetti, 1991; Ehri, 1992; Seymour, 1994; Ans et al., 1998; Grainger et al., 2012). The acquisition of stable GP mappings and accurate knowledge of letters’ position in the word would be necessary conditions for the strengthening of words’ orthographic representation allowing its automated recognition (Perfetti, 1992). Indeed, repeated correct phonological recoding of a written word allows to store the words’ representation in the orthographic lexicon (self-teaching hypothesis; Share, 1995). Finally, training with GP mappings seems to induce a refinement of phonological awareness, known as a crucial predictor of reading acquisition (Perfetti et al., 1987; Goswami, 1993).

Several methods have been developed for teaching to read, and many teachers use at least two different methods in parallel (“mixed” approach) (Deauvieau and Terrail, 2018). The first method, which we refer to here as “phonics,” involves explicit and progressive teaching of GP mappings, through a variety of exercises and items, allowing transfer to new letter strings. The second method, which we refer to as “global,” involves teaching of a strict visual recognition strategy (visual memorization of the whole word) in order to create a direct mapping between the written word, its spoken form, and its meaning.

Behavioral classroom studies have shown that the type of method has a higher impact on children’s future reading performances than other variables (e.g., socio-economic background, performances in kindergarten, teachers’ experience) (Braibant and Gerard, 1996; Goigoux, 2000; Deauvieau et al., 2013). These studies and meta-analyses (Ehri et al., 2001; Rayner et al., 2001) conclude that alphabetical approaches are more effective than mixed or non-alphabetical approaches in word reading, spelling, and in text comprehension (Deauvieau and Terrail, 2018). They also induce the highest improvements in children at risk of a reading disorder or with low socio-economic background, by increasing their self-teaching ability (Ehri et al., 2001; Rayner et al., 2001; Goigoux, 2016). On the contrary, mixed or non-alphabetical approaches give rise to the highest proportion of poor readers and generate a higher heterogeneity of performance within a class (Braibant and Gerard, 1996; Goigoux, 2000; Deauvieau et al., 2013). In agreement with the self-teaching hypothesis (Share, 1995), sole visual exposure or limited phonological recoding (e.g., concurrent articulation) significantly reduces orthographic learning of novel letter strings (Share, 1999; Kyte and Johnson, 2006; De Jong et al., 2009). Also, kindergarten children trained to memorize an artificial script or one-syllable words with a global strategy, have a lower ability to read novel stimuli than children trained with a GP mapping strategy (Jeffrey and Samuels, 1967) and have difficulties to infer GP mappings that have not been trained explicitly (Byrne, 1991, 1996).

Adults’ behavioral studies have confirmed the lower efficiency of the global method concerning the transfer to novel stimuli and the implicit acquisition of GP mappings (Bishop, 1964; Byrne, 1984; McCandliss et al., 1997; Bitan and Karni, 2003; Yoncheva et al., 2010). Studies using functional magnetic resonance imaging (fMRI) with unfamiliar stimuli (e.g., artificial script, pseudowords) have shown that in comparison to global training (e.g., visual shape recognition, direct mapping with meaning) training based on phonological recoding leads to a response modulation in the L-VOTC (Sandak et al., 2004; Xue et al., 2006). Furthermore, the specific involvement of the VWFA in mapping print to phonology has been demonstrated in a study which contrasted the association of an artificial script with speech sounds and non-speech sounds (Hashimoto and Sakai, 2004). Studies using electroencephalography (EEG) showed that training GP mappings led to a left-lateralized visual evoked potential (N170) response sensitive to trained and untrained artificial script (between subjects design: Yoncheva et al., 2010; within-subjects design: Yoncheva et al., 2015), while global training led instead to a right-lateralized N170 response. Collectively, these observations highlight that the unit’s size on which the learner focuses, and therefore the processes engaged in word recognition after learning, directly influence neural processes. They also suggest that the phonics method engages the typical left lateralized brain circuitry of reading, while the global method based on rote-learning of visual forms, engages relatively more the right hemisphere.

However, how (different) teaching methods impact brain activity in *children* engaged in formal reading acquisition remains currently unknown. The current study aims at filling this gap in the context of a mixed approach, by comparing neural responses in children to letter strings processed by GP mappings (French control words and pseudowords) vs. to letter strings that have been rote-learned (French global words) and could therefore trigger whole word-form visual recognition processes. Given the above-mentioned negative outcomes of the global method for developing reading ability, children were not *trained* with different teaching methods (global vs. phonics). Rather, we built our experimental material on the basis of the reading acquisition methods implemented in classrooms. That is, at a specific time of the school year, in the mixed approach used by teachers, some words have been taught with a global method, whereas the phonics method had been used to teach GP mappings with other words. More precisely, in the two schools where the study was conducted, a list of global words was provided by the teachers, from which we extracted a common sub-part of twenty 4- and 5-letters words. These words have been globally taught in classrooms (mapping of whole-visual word form to spoken form, without knowledge of the inner GP mappings), then printed on small cards given to the children in the so-called “words’ box,” and home-trained every day. In parallel, teachers taught GP mappings at the rate of one letter per week, with a variety of exercises: write the letter, recognize it within words, learn its case variants, etc. At the time of our testing, 9 letters have been taught in classrooms (a, é, e, è, i, o u, r, l). The other letters have been encountered by children and were variably recognized (as attested by performances in behavioral tests, see Table 1).

**TABLE 1:** Behavioral scores for the cognitive and reading assessment for the whole group (*N* = 42) and per subgroup.

	**Scores: mean (SD)**			**Independent *t*-tests**	
		
**Behavioral tests and sub-tests**	**Total *N* = 42**	**Good readers *N* = 18**	**Poor readers *N* = 19**	***t*-Value**	***p*-Value**
**General cognitive functions**					
Non-verbal intelligence (CPM,% accuracy)	76.26 (12.20)	83.33 (8.83)	69.44 (11.26)	4.157	0.000
Selective attention (TEA-Ch, speed in sec)	6.75 (2.36)	5.91 (2.29)	7.58 (2.41)	−2.154	0.038
Vocabulary production (N-EEL,% accuracy)	77.63 (9.57)	84.70 (5.34)	71.28 (8.89)	5.523	0.000
**Reading ability**					
Single letters (BELO,% accuracy)	71.43 (20.48)	88.46 (7.22)	54.45 (17.45)	7.667	0.000
Composite score (BELO, BALE,% accuracy)	34.57 (18.75)	51.67 (11.54)	18.36 (10.27)	9.253	0.000

**FIGURE 1:**
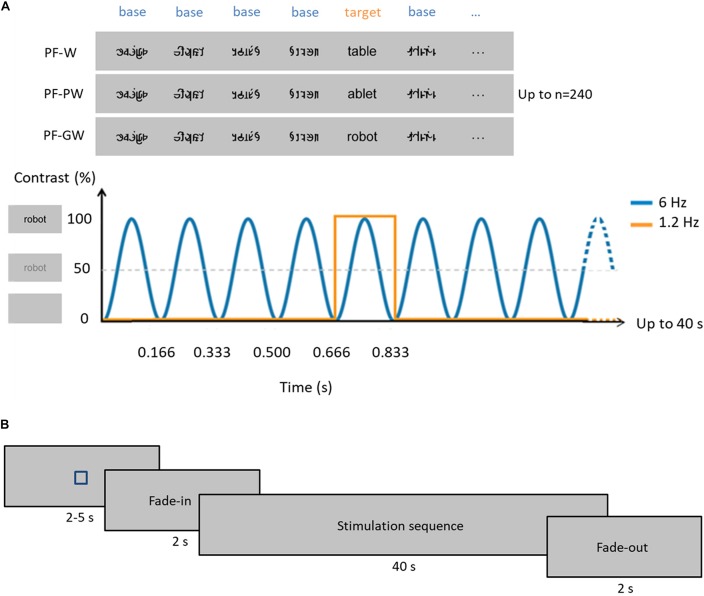
Experimental paradigm. **(A)** In each condition, base stimuli were pseudofonts (top, middle, and bottom rows), and target stimuli were either control words (W, top row), pseudowords (PW, middle row) or globally taught words (GW, bottom row) appearing every fifth item. Each sequence lasted 40 s, during which stimuli were presented by sinusoidal contrast modulation at 6 Hz, each stimulus reaching full contrast after 83 ms (i.e., one cycle duration = 166.66 ms). Stimulation alternated between base (B) and target (T) stimuli such as BBBBTBBBBTBBB. Target stimuli therefore appeared at 6 Hz/5, so at 1.2 Hz. Stimuli were randomly presented with no immediate repetition and appeared continuously on the screen. In total, 240 stimuli were presented per sequence (48 target stimuli and 192 base stimuli), and each condition was repeated three times. **(B)** Timeline of a sequence: each sequence started with a fixation square (for 2–5 s) after which the stimulation faded in (for 2 s) then reached full contrast (for 40 s) and then faded out (for 2 s) (see section “Materials and Methods”).

To assess neural responses in children, we used a recently developed Fast Periodic Visual Stimulation approach with electroencephalographic recordings (FPVS-EEG). In this approach, our stimuli of interest (**control words**, **control pseudowords**, or **global words**; **W**, **PW**, **GW** hereafter) are inserted periodically every five items within a rapid stream of base stimuli (6 Hz) (Figure 1), that were constituted of pseudofonts. Given its high sensitivity (high Signal-to-Noise Ratio, SNR) (Regan, 1989; Norcia et al., 2015), FPVS-EEG allows to rapidly (i.e., in a few minutes) measure selective responses to rare/deviant stimuli. Recently, robust discrimination response for words within pseudofonts, non-words, or even pseudowords was shown in adults over the left occipito-temporal cortex (Lochy et al., 2015). In 5 years-old preschoolers, a letter-selective discrimination response (words/pseudowords within pseudofonts) was observed over left posterior regions of the scalp and, critically, correlated with GP knowledge (Lochy et al., 2016). These findings revealed the potential of the FPVS-EEG approach to assess the neuro-cognitive representations of written words and the development of neural circuits for reading (see also Lochy et al., 2018 for intracerebral evidence). Furthermore, as a behavior-free (implicit and automatic visual discrimination of letter strings) and highly sensitive approach, FPVS-EEG is ideal to test young children.

In the current study, we first hypothesized that control W/PW would trigger left-lateralized responses in agreement with the above-reviewed literature, more specifically with the phonological mapping hypothesis (Maurer and McCandliss, 2007), and also with previous findings using the same experimental paradigm showing a relationship between letter knowledge and left-lateralized responses (Lochy et al., 2016).

Second, we hypothesized that GW, if recognized by a whole-word form visual recognition process, would display a different lateralization pattern than letter strings processed by GP mappings (W/PW). That is, we expected that responses to GW should be bilateral or right-lateralized, as observed in adults with artificial script (Yoncheva et al., 2010, 2015).

Here, in the context of a mixed approach, the children have learnt both processes (GP mappings and visual recognition) and GW might also be processed by GP mappings. Therefore, we also postulated that letter knowledge (or early reading ability) would influence the reliance on one or the other process for GW. Indeed, poor readers having difficulties to automatize GP mappings (e.g., unstable knowledge of letters’ sounds (Perfetti, 1992), deficits in phonological awareness (Ziegler et al., 2010) and/or visuo-attentional processing (Ans et al., 1998), compensate their difficulties by using alternative strategies for reading (e.g., salient visual features; Campbell and Butterworth, 1985; Vellutino, 1987). Thus, we hypothesized that the comparison of children who know more or fewer letters might reveal a differential effect for those letter strings that can be processed by relying essentially on visual recognition, i.e., GW. If the children with weaker letter knowledge rely predominantly on visual recognition whenever possible (i.e., for GW), then they should show either less responses in the left hemisphere (Lochy et al., 2016), and/or more responses in the right hemisphere (Yoncheva et al., 2010, 2015), for GW as compared to control W or PW. This is not expected from children who have automatized GP mappings and may transfer this process on all types of letter strings, including GW.

Finally, the comparison of groups of children within our sample might also help disentangle the potential role of familiarity, intrinsically linked with the two different methods and stimuli used. Since all children have been familiarized explicitly and to the same degree with the GW, group differences regarding responses to GW would strongly suggest that familiarity *per se* is not a key factor. Indeed, if familiarity induces engagement of the right hemisphere, known to be preferentially involved in visual object and face recognition Farah, 1990; Rossion et al., 2003), then this should be the case irrespective of the children’s letter knowledge.

## Materials and Methods

### Participants

First grade children (*N* = 44) from two Belgian schools (20 boys, mean age = 6 years, 5 months; range = 5 years, 11 months-7 years, 11 months, 41 right-handed) were tested in the first trimester of grade 1 after the parents gave a written informed consent for a study approved by the Biomedical Ethical Committee of the Université catholique de Louvain. Two children were excluded because of abnormal performances in behavioral tests (see below). All had normal or corrected-to-normal vision. They were unaware of the goal of the study and that a change of stimulus type occurred at a periodic rate during stimulation. The testing took place in a quiet room of the school in two or more sessions (EEG, behavioral).

### Behavioral Testing

General cognitive functions and reading ability were assessed by means of standardized tests and subtests: non-verbal intelligence (CPM; Raven, 1998), selective attention (TEA-Ch; Manly et al., 2004), vocabulary production (N-EEL; Chevrie-Muller and Plaza, 2001) and reading of single letters, syllables, regular words, irregular words, pseudowords (BELO; George and Pech-Georgel, 2012, BALE; Jacquier-Roux et al., 2010). Individual z scores were computed in order to identify outliers within the distribution of the current sample. One child was excluded because of scores lower than 2 standard deviations in all general cognitive functions and another one was excluded because of a medicated attentional disorder. Descriptive statistics of included children (Table 1) highlight a great heterogeneity of reading scores within the group (see Supplementary Table S2).

### EEG Testing

#### Stimuli

Four categories of 20 stimuli were used for this experiment (Figure 1): pseudofonts (PF), French words taught with a global method at school (GW) and control French words (W) or pseudowords (PW). The natural school context of learning provided us with the “global words.” We chose 4- and 5-letters words (*N* = 10 of each) from the “words’ box” that teachers had provided children at the beginning of the school year and which contained the words learnt item by item by mapping the whole-word form to its phonological counterpart. Control words were words for which children did not receive any explicit instruction and were selected from the Manulex database (Lété et al., 2004) to match global words in lexical frequency, bigram frequency, orthographic neighborhood density and in number of letters (four or five). W and GW did not differ in *frequency estimated for grade 1* [*t*(38) = −0.89; *p* = 0.380; *W* = 79.40 ± 38.98 SD, GW = 102.00 ± 106, 96 SD], *standard frequency index* [*t*(38) = 0.60; *p* = 0.552; *W* = 65.35 ± 2.68 SD, GW = 64.56 ± 5.20 SD], *estimated frequency of use* [*t*(38) = −0.69; *p* = 0.495; *W* = 400 per million ± 197.68 SD, GW = 486 per million ± 525.18 SD], *bigram frequency* [*t*(38) = −0.36; *p* = 0.971; *W* = 8390.10 ± 4261.80 SD, GW = 8440.95 ± 4601.25 SD] or *orthographic neighborhood density* [*t*(38) = 0.73; *p* = 0.467; *W* = 5.55 ± 4.46 SD, GW = 4.50 ± 4.57 SD]. Pseudowords were pronounceable letter strings which respected the phonological rules in French. They were built one by one on the basis of the words by changing the position of their constitutive letters (e.g., the words “page” and “table” give rise to the pseudowords “gape” and “ablet”). Pseudowords were matched with all words (W and GW) in bigram frequency, identity of letters and in number of letters (four or five). Pseudowords did not differ in *bigram frequency* (8141.15 ± 3491.40 SD) from words [8390.10 ± 4261.80 SD; *t*(38) = 0.20; *p* = 0.841] or global words [8440.95 ± 4601.25 SD; *t*(38) = 0.23; *p* = 0.818]. A complete list of stimuli used is provided in Supplementary Table S1. After the experiment, we assessed how many of the letters used in the control letter strings were recognized during the behavioral letter recognition task. On average, children knew 14/18 (mean accuracy = 79.37% ± 19.80%, minimum = 27.78%, maximum = 100%) letters used in our stimuli (60% of the children knew 15/18 letters or more). Pseudofont stimuli were also built one by one on the basis of all the words (W and GW): each word was vertically flipped and its letters were segmented into simple features by using Adobe Photoshop. These segments were then rearranged to form pseudoletters, respecting the total number of characters (four or five) and the overall size (width × height) of the original word (Lochy et al., 2015, 2016). Pseudoletters thus contained junctions, ascending/descending features and close-up shapes. Therefore, each word (W or GW) had a corresponding pseudoword and pseudofont, containing the exact same amount of black-on-white contrast, so that all conditions were comparable in terms of low-level visual properties.

These different stimuli allowed to create three conditions (Figure 1). In each condition, base stimuli were pseudofonts (PF), and target stimuli were either global words (PF-GW condition), control words (PF-W condition) or pseudowords (PF-PW condition). Stimuli were presented centrally in Verdana font with a height between 47 and 77 pixels and a width between 103 and 271 pixels, depending on the shape of the individual letters. At a viewing distance of 1 m with a screen resolution of 800 × 600 pixels and a refresh rate of 60 Hz, stimuli ranged from 2.69 to 7.07 (width) and 1.32 to 2.18 (height) degrees of visual angle.

#### Procedure

The stimulation procedure was very similar to previous FPVS-EEG studies on word recognition (Lochy et al., 2015, 2016). Each stimulation sequence started with a fixation square displayed for 2–5 s (randomly jittered between sequences), 2 s of gradual stimulation fade-in, 40 s of stimulation, and 2 s of fade-out. Stimuli were presented by means of sinusoidal contrast modulation at a base frequency rate of 6 Hz with Java (SE Version 8) (i.e., one item every 166.66 ms, from a gray background to full contrast and back in 166.66 ms thus, each item reached full contrast at 83 ms). Every fifth stimulus (1/5) of the sequence (frequency of 1.2 Hz thus, every 833 ms), a global word (PF-GW sequence) or a control letter strings (PF-W or PF-PW sequences) was presented. Each condition was repeated three times. Considering a total of 40 s (sequence duration) × 3 (repetitions) × 3 (conditions), 6 min of stimulation were presented in total, plus 48 s of fade-in and fade-out (Figure 1B). There was a pause between each sequence, which was initiated manually to ensure low-artifact EEG signals, and where the child was proposed a rest if needed. Altogether, the testing lasted 10–15 min depending on the child, including breaks.

During the stimulation, children continuously fixated a central square and were instructed to press the space bar upon any brief (200 ms) color change of the fixation square (blue to yellow; six changes randomly timed per sequence) (see Supplementary Video S1). This orthogonal task was included to maintain both a central eyes position on the screen and a constant level of attention throughout the entire stimulation (see Lochy et al., 2015), and was performed almost at ceiling (95.93 ± 6.46% SD accuracy). There were no significant differences between conditions with respect to accuracy [*F*(2,78) < 1], or response time [*F*(2,78) < 1].

#### Acquisition

During EEG recording, children were seated comfortably in a quiet room in the school at a distance of 1 m from the computer screen. EEG signal was acquired at 1.024 Hz by using a 32-channel Biosemi Active II system (Biosemi, Amsterdam, Netherlands), with electrodes including standard 10–20 system locations. The magnitude of the offset of all electrodes, referenced to the common mode sense, was held below 50 mV.

#### Preprocessing

All EEG analyses were carried out by using Letswave 5.c^1^ and Matlab 2014 (The Mathworks) and followed procedures validated in several studies using letter strings or faces and objects stimuli (see, e.g., Retter and Rossion, 2016). After band-pass filtering between 0.1 and 100 Hz, EEG data were segmented to include 2 s before and after each sequence, resulting in 44 s segments. Data files were then downsampled to 256 Hz to reduce file size and data processing time. Artifact or noisy channels were replaced by using linear interpolation. All channels were re-referenced to the common average. EEG recordings were then segmented again from stimulation onset until 39.996 s, corresponding exactly to 48 complete 1.2 Hz cycles, which is the largest amount of complete cycles of 833 ms at the target frequency (1.2 Hz) within the 40 s of stimulation period.

#### Frequency Domain Analysis

To reduce EEG activity that is not phase-locked to the stimulus, the three repetitions of each condition were averaged in the time domain for each individual participant. Then, to convert data into the frequency domain, a Fast Fourier Transform (FFT) was applied to these averaged time windows and normalized amplitude spectra were extracted for all channels. This procedure yields EEG spectra with a high frequency resolution (1/39.996 s = 0.025 Hz), increasing SNR (Regan, 1989; Rossion, 2014), allowing unambiguous identification of the response at the exact frequencies of interest (i.e., 6 Hz and its harmonics for the base stimulation rate and 1.2 Hz and its harmonics for the target stimulation rate). All of the responses of interest, and thus all the potential differences between conditions, can be concentrated in a discrete frequency band around the stimulation frequency. This frequency band occupies a very small fraction of the total EEG bandwidth. In contrast, biological noise is distributed throughout the EEG spectrum, resulting in a SNR in the bandwidth of interest that can be very high (Regan, 1989; Rossion, 2014). To estimate SNR across the EEG spectrum, amplitude at each frequency of interest (bin) was divided by the average amplitude of 20 surrounding bins (10 on each side) (Liu-Shuang et al., 2014).

To quantify the responses of interest in microvolts, the average voltage amplitude of the 20 surrounding bins (i.e., the noise) was subtracted out (e.g., Dzhelyova and Rossion, 2014; Retter and Rossion, 2016) (baseline-subtracted amplitudes). To assess the significance of the responses at the target frequency and harmonics, and at the base rate and harmonics, z scores were computed at every channel on the grand averaged amplitude spectrum for each condition (e.g., Liu-Shuang et al., 2014; Lochy et al., 2015). z scores larger than 2.58 (*p* < 0.01, one-tailed, signal > noise) were considered significant. A conservative threshold was used because the response was evaluated on all channels (although we expected responses at posterior channels), and distributed on several harmonics as in previous studies using the same approach with letter strings (Lochy et al., 2015, 2016) or faces (Liu-Shuang et al., 2014). An identical number of harmonics was selected across all conditions and electrodes based on the condition in which the highest number of consecutive harmonics was significant on any electrode (in total, 4 harmonics for target responses and 6 harmonics for base responses). Finally, to quantify the periodic response distributed on several harmonics, the baseline subtracted amplitudes of significant harmonics (except the base stimulation frequency) were summed for each participant (see Retter and Rossion, 2016 for validation of the procedure).

## Results

### Whole Group Analysis

#### Letter Discrimination Responses

Selective discrimination responses to letter strings (W, PW, GW) inserted in pseudofonts were significant (z scores > 2.58) at exactly 1.2 Hz and several harmonics at several electrodes. As determined by grand-averaged data (see section “Materials and Methods”), the highest number of consecutive significant harmonics were four (from F/5 or 1.2 Hz to 4F/5 or 4.8 Hz). In order to determine the electrodes of interest, all electrodes were then ranked according to their largest amplitude values for the sum of baseline subtracted amplitudes computed on four significant harmonics (see section “Materials and Methods”). In all conditions, most of the response was captured on the left occipital channel O1 (= 2.35 μV), the EEG amplitude decreasing sharply on nearby electrodes (P7 = 1.73 μV; PO3 = 1.07 μV; P3 = 0.13 μV). Therefore, in line with a previous study in children with the same approach (Lochy et al., 2016), we focused on O1 and its homologous right hemispheric electrode O2.

**FIGURE 2:**
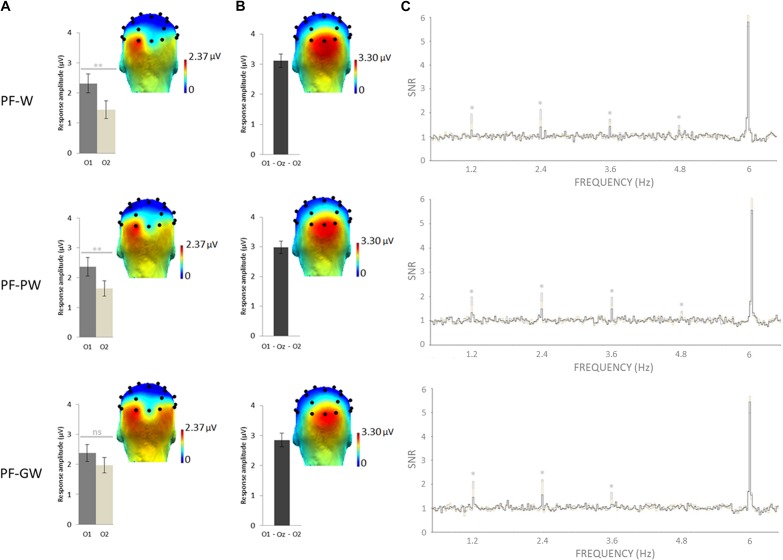
Response amplitudes and topographies of the whole group. Grand-averaged (*N* = 42) scalp topographies for the response in each condition at **(A)** the target and **(B)** base frequencies (sum of baseline subtracted amplitudes at significant harmonics; see section “Materials and Methods”). Histograms represent the same data, with standard errors of the mean. For letter strings-selective responses, stars indicate significant difference between the left (O1) and the right (O2) occipital electrodes (^∗∗^*p* < 0.01, ns: not significant). **(C)** SNR EEG spectra on O1 (dark gray), O2 (in light gray), and Oz (in black) for each condition, stars indicate significant responses at letter strings-selective frequency and harmonics (1.2, 2.4, 3.6, and 4.8 Hz). Individual EEG data summarized in Figure 2 are provided in Supplementary Data Sheets S2–S4, and described in Supplementary Data Sheet S1.

An ANOVA was performed on target discrimination responses (sum of baseline subtracted amplitudes) with *Hemisphere* (left-O1, right-O2) and *Condition* (PF-W, PF-PW, PF-GW) as within-subjects factors. It revealed a main effect of *Hemisphere* [*F*_1_,_41_ = 9.24, *p* = 0.004, η^2^ = 0.20], no effect of *Condition* [*F*_2_,_82_ = 0.82, *p* = 0.445, η^2^ = 0.02] and a marginally significant interaction between these two factors [*F*_2_,_82_ = 3.08, *p* = 0.05, η^2^ = 0.07]. Paired samples *t*-tests were performed in order to compare the response amplitude between the left (O1) and the right (O2) hemispheres in each condition. Response amplitude to both control words (O1 = 2.31 μV, O2 = 1.44 μV) and pseudowords (O1 = 2.37 μV, O2 = 1.64 μV) was stronger in the left (O1) than in the right (O2) hemisphere (PF-W: [*t*(41) = 3.45; *p* = 0.001]; PF-PW: [*t*(41) = 3.16; *p* = 0.003]), but response amplitude to global words did not significantly differ between the left (O1 = 2.37 μV) and the right (O2 = 1.97 μV) hemispheres (PF-GW: ([*t*(41) = 1.59; *p* = 0.120]) (Figure 2). We also ran an ANOVA with *Hemisphere* (O1, O2) and *Conditions* (PF-W, PF-PW, PF-GW) with a split sample as a function of gender. The results were similar for boys and girls, except that boys were less left-lateralized than girls *in all conditions*, given that this factor did not reach significance in boys [*F*(1,19) = 2.513; *p* = 0.12; η^2^ = 0.12], while it did in girls (*F*(1,21) = 8.584; *p* = 0.008; η^2^ = 0.29) (see also section “Analyses of Gender Effects” in the Supplementary Data Sheet S5).

#### Base Rate Responses

The base stimulation frequency reflects the neural synchronization to the general visual periodic stimulation at 6 Hz. z scores computation revealed significant responses in all conditions at exactly 6 Hz and several harmonics on middle occipital electrodes. As determined by grand-averaged data (see “Materials and Methods”), the highest number of consecutive significant harmonics were six (from F or 6 Hz to 6F or 36 Hz). In order to determine the electrodes of interest, all electrodes were then ranked according to their largest amplitude values for the sum of baseline subtracted amplitudes computed on six significant harmonics (see section “Materials and Methods”). In all conditions, the largest response was recorded at three middle occipital (MO) electrodes: O1 (2.72 μV), O2 (3.04 μV) and Oz (3.18 μV). Therefore, we averaged their amplitude values for analyses (MO ROI = mean O1, Oz, O2) (Figure 2). An ANOVA performed on response amplitudes in MO ROI with *Condition* (PF-W, PF-PW, PF-GW) as within-subjects factor, did not reveal any effect of *Condition* [*F*_2_,_82_ = 1.26, *p* = 0.288, η^2^ = 0.03].

### Analysis by Reading Level

We computed a composite score of reading for each child by averaging accuracy scores for single letters, syllables, pseudowords and words reading. Since our objective was to compare subgroups, we assigned children in subgroups on the basis of the group’s mean composite score (34.57% of accuracy). Children who performed above the group’s mean composite score (>35%) were considered as “good readers” (*N* = 18; 10 boys), and those below (<34%) as “poor readers” (*N* = 19; 7 boys). The two groups did not differ in gender distribution [*Chi2* (1,37) = 1.303; *p* = 0.254; phi = 0.18]. We excluded from the analysis 5 children whose score was at the group’s mean composite score (34–35%). Results of each subgroup for reading and general cognitive assessment are displayed in Table 1. Concerning the orthogonal task of color-change detection, an ANOVA performed on response times with *Condition* (PF-W, PF-PW, PF-GW) as within-subjects factor and *Group* (Good readers, Poor readers) as a between-subjects factor, showed no main effect or interaction (all *F*s < 1).

#### Letter Discrimination Responses

**FIGURE 3:**
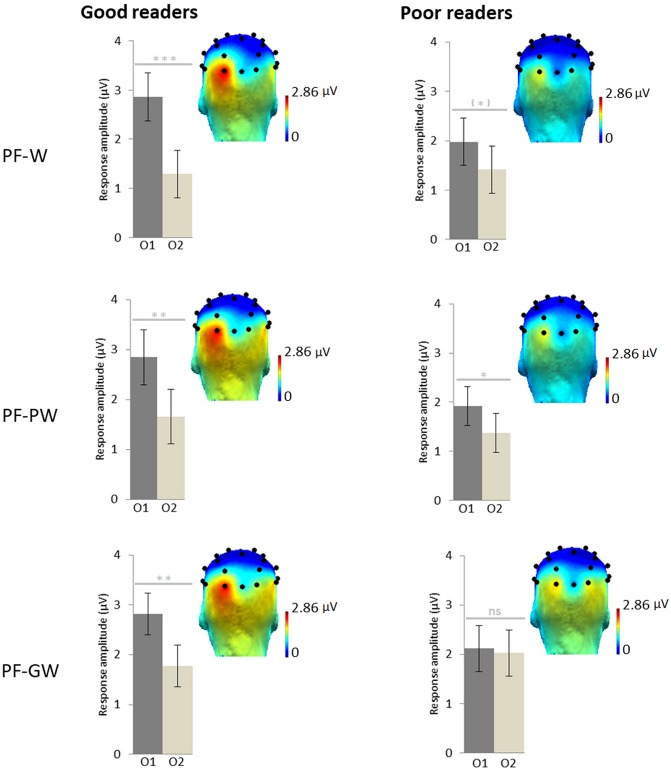
Response amplitudes and topographies by reading level. Scalp topographies for the response in each condition at the target frequency (sum of baseline subtracted amplitudes at significant harmonics: 1.2, 2.4, 3.6, and 4.8 Hz; see section “Materials and Methods”) for children assigned in the good readers group (*N* = 18) and in the poor readers group (*N* = 19) on the basis of their behavioral reading scores. Histograms represent the same data, with standard errors of the mean. Stars indicate significant difference between the left (O1) and the right (O2) occipital electrodes (^∗^*p* < 0.05; ^∗∗^*p* < 0.01; ^∗∗∗^*p* < 0.001, ns: not significant). Individual EEG data summarized in Figure 3 are provided in Supplementary Data Sheets S2–S4, and described in Supplementary Data Sheet S1.

An ANOVA was performed on target discrimination responses (sum of baseline subtracted amplitudes) with *Hemisphere* (left-O1, right-O2) and *Condition* (PF-W, PF-PW, PF-GW) as within-subjects factors and *Group* (good readers, poor readers) as between-subjects factor. It revealed an effect of *Hemisphere* [*F*_1_,_35_ = 18.38, *p* = 0.000, η^2^ = 0.34], responses being overall stronger in the left hemisphere (O1 = 2.41 μV, O2 = 1.59 μV) and most importantly a significant interaction between *Hemisphere* and *Group* [*F*_1_,_35_ = 4.96, *p* = 0.033, η^2^ = 0.12]. The interaction was due to responses of the two groups being almost identical in the right hemisphere (good readers: 1.60 μV, poor readers: 1.57 μV), while good readers had a stronger response than poor readers in the left hemisphere (2.84 and 2.01 μV respectively). There was also a non-significant trend for an interaction between *Hemisphere* and *Condition* [*F*_2_,_70_ = 2.89, *p* = 0.063, η^2^ = 0.08] but no main effect of *Condition*, *Group* or any other interaction (all *F*s < 1). Given our *a priori* hypothesis of a modulation of the effects by reading level, and the interactions *Hemisphere × Group* and *Hemisphere × Condition*, as well as the trends highlighted by the topographies and the histograms (Figure 3), paired *t*-tests were performed between O1 and O2 in each condition and group. Good readers presented a significantly stronger response in the left hemisphere in each condition; PF-W: [*t*(17) = 4.53; *p* = 0.000] (O1 = 2.86 μV, O2 = 1.29 μV), PF-PW: [*t*(17) = 3.67; *p* = 0.002] (O1 = 2.84 μV, O2 = 1.66 μV), PF-GW: [*t*(17) = 2.93; *p* = 0.009] (O1 = 2.82 μV, O2 = 1.78 μV). Poor readers presented a trend for left lateralized response in PF-W [*t*(18) = 1.80; *p* = 0.088] (O1 = 1.98 μV, O2 = 1.42 μV), a left lateralized response in PF-PW [*t*(18) = 2.21; *p* = 0.040] (O1 = 1.92 μV, O2 = 1.38 μV) and a bilateral response in PF-GW [*t*(18) = 0.26; *p* = 0.802] (O1 = 2.12 μV, O2 = 2.03 μV).

#### Base Rate Responses

An ANOVA performed on response amplitudes in MO ROI with *Condition* (PF-W, PF-PW, PF-GW) as within-subjects factor and *Group* (good readers, poor readers) as between-subject factor, revealed no effect of *Condition* [*F*_2_,_70_ = 1.15, *p* = 0.320, η^2^ = 0.03], no effect of *Group* [*F*_1_,_35_ = 3.52, *p* = 0.07, η^2^ = 0.09] and no interaction between these two factors [*F*_2_,_70_ = 2.24, *p* = 0.114, η^2^ = 0.06].

### Brain-Behavior Correlations

**FIGURE 4:**
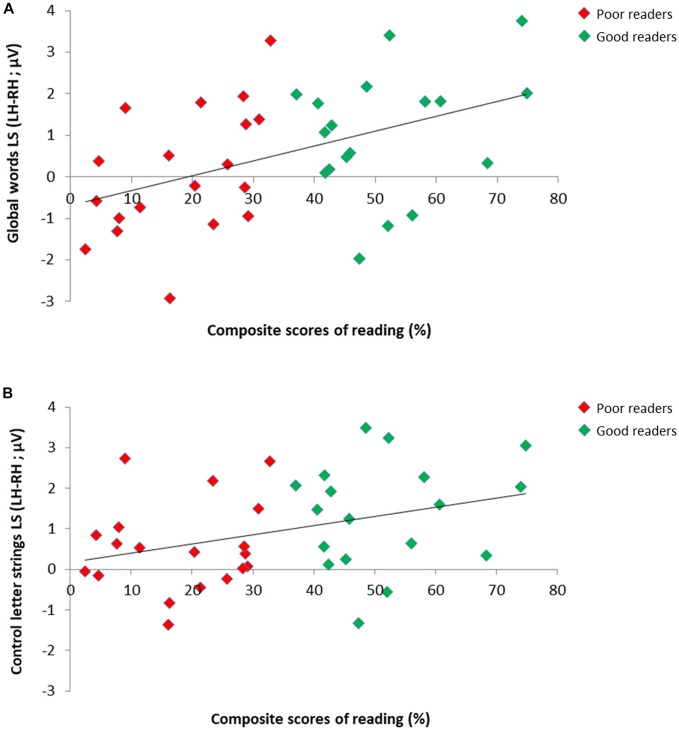
Relation between individual reading scores and EEG lateralization scores for the responses to letter strings in 37 children. Scatter plots of significant positive correlation between composite scores of reading (averaged accuracy scores for single letters, syllables, pseudowords and words) and EEG lateralization scores (LH-RH) for the responses to **(A)** global words (Spearman Rho = 0.44) and **(B)** control letter strings (averaged response to control words/pseudowords; Spearman Rho = 0.36) in good readers (*N* = 18; green dots) and poor readers (*N* = 19; red dots).

We assessed if reading scores correlated with lateralization scores (LS; calculated as LH–RH) for the responses to letter strings on the 37 children retained in our subgroups analysis (Figure 4), and this, separately for the response to GW, W, PW, as well as the average of W/PW (as in Lochy et al., 2016). All these correlations were significant (respectively: Spearman Rho = 0.44, *p* = 0.003; Rho = 0.43, *p* < 0.004; Rho = 0.29, *p* = 0.043; Rho = 0.36, *p* = 0.016), indicating that better readers tended to reveal more left-lateralized responses in all conditions.

**FIGURE 5:**
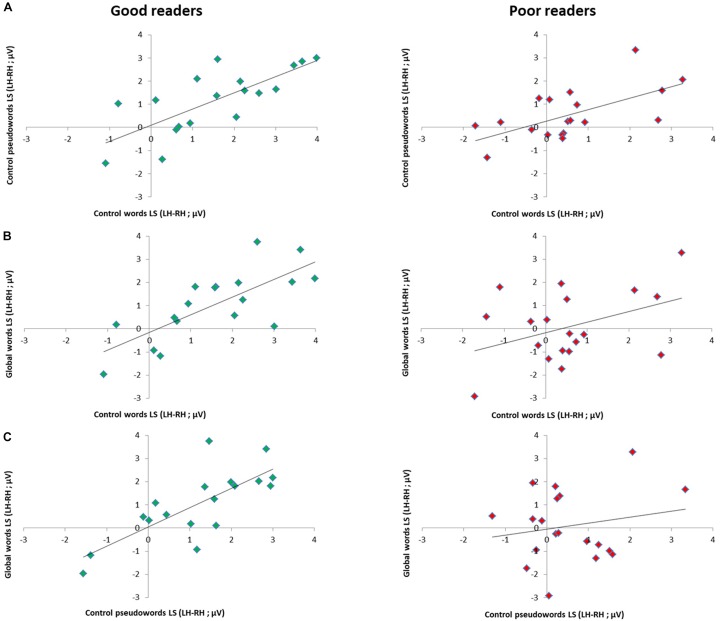
Relation between EEG lateralization scores (LS) across conditions per reading level subgroup. Scatter plots of correlation analysis between EEG lateralization scores (LH-RH) for the responses to **(A)** control words and control pseudowords, **(B)** global words and control words and to **(C)** global words and control pseudowords in good readers (*N* = 18, green dots) and poor readers (*N* = 19, red dots). Correlations were all highly significant in good readers (all *p* < 0.001), while in poor readers, responses to global words did not correlate with control letter strings (with W: *p* = 0.22; with PW: *p* = 0.44), which correlated together (*p* = 0.002), suggesting atypical processing of global words.

Per subgroup, we computed correlations between conditions, reasoning that if distinct processes are triggered for GW, then LS should not correlate with the other conditions. In the good readers group, all conditions correlated highly: W and PW (Spearman Rho = 0.79; *p* = 0.000), W and GW (Spearman Rho = 0.78; *p* = 0.000), PW and GW (Spearman Rho = 0.76; *p* = 0.000). In the poor readers group, only W and PW correlated significantly (Spearman Rho = 0.64; *p* = 0.002), while GW did not correlate with the two other conditions (with W and PW, respectively Spearman Rho = 0.19; *p* = 0.22; and Rho = 0.035; *p* = 0.44) (Figure 5).

## Discussion

In the context of a mixed approach for teaching to read, our study reveals that distinct phonics/global methods differentially impact neural responses to letter strings in children, suggesting that the qualitatively different cognitive processes triggered by each of these methods (GP mappings vs. holistic visual recognition) rely on different neural networks. On the whole sample (*N* = 42), results show that words taught globally are processed more bilaterally than pseudowords or words that have not been taught with this whole-word visual form recognition. However, our results also suggest that reading ability may modulate this effect: children who knew only few GP mappings (“poor readers”) indeed process global words differently (bilaterally) than control words/pseudowords, while children who knew more GP mappings (“good readers”) engaged the left hemisphere (LH) relatively more, irrespective of the type of letter string. We discuss these points in turn below.

We compared neural responses to three categories of stimuli for which different processing types were hypothesized. First, control pseudowords and control words should trigger decoding/conversion procedures and hence rely on GP mappings knowledge. Second, words that have been taught globally could be processed either by recalling rote knowledge of the visual form, or by decoding abilities. At the whole group level, our results showed that GP mappings predominantly engaged the LH while whole-word recognition engaged also the right hemisphere (RH). The overall left lateralization in response to letter strings that are decoded (words/pseudowords) in beginning readers confirms recent findings (FPVS-EEG, Lochy et al., 2016; fMRI, Dehaene-Lambertz et al., 2018) and supports the Phonological Mapping Hypothesis (Maurer and McCandliss, 2007) proposing that simple passive viewing of letter strings triggers activation of associations between orthographic and phonological representations (Maurer et al., 2006; Karipidis et al., 2018; Pleisch et al., 2019). Remarkably, knowledge of all letters is not necessary to observe this LH lateralization pattern in response to letter strings. In the current study, stimuli were constituted of 18 different letters, of which only 9 had been formally taught with the phonics method at school, and on average 14 were known. Similarly, in a previous kindergarten study (Lochy et al., 2016), children who knew more than 9 letters already showed this typical LH pattern of responses to letter strings. This first finding therefore confirms that triggering of GP mappings induces LH dominant responses. It also replicates the finding that children who know more letters (“good readers”) have stronger responses in the LH than children who know fewer letters (“poor readers”) (Lochy et al., 2016). On the other hand, words learnt globally, thus with a rote association between the visual form and the spoken form, induce bilateral responses.

Our data agree with previous findings in adults trained with an artificial script. In these studies, globally learnt stimuli engaged the RH more than items learnt with a phonics approach (Yoncheva et al., 2010, 2015). The RH is known to preferentially support holistic visual recognition, in particular of face stimuli (Farah, 1990; Rossion et al., 2003), and it has been suggested to reflect visual familiarity with script in tasks where a minimal visual recognition strategy was possible, for instance in a one-back task (Maurer et al., 2010). At a broader level, the two hemispheres are hypothesized to preferentially support different types of processes, the LH showing an advantage for analytic/local processes while the RH shows an advantage for holistic/global processes (Sergent, 1982; Corballis, 2003), as evidenced by neuropsychological deficits of patients with unilateral brain damage (reviewed in Ivry and Robertson, 1998).

We had hypothesized, based on behavioral studies, that reading level might modulate the reliance on visual strategies and hence a differential involvement of LH and RH, because children who know fewer letters (hence had less memorized/automatized GP mappings) show the tendency to develop alternative strategies for reading (Campbell and Butterworth, 1985; Vellutino, 1987). Although we did not find a significant *Group × Hemisphere × Condition* interaction, our data nevertheless show trends in this direction that cannot be ignored, because of their important implication for education. Analyzing groups and conditions separately showed that “poor readers” have a lack of left lateralized response to global words, contrary to children who knew more letters. This finding suggests that poor readers relied more on visual recognition processes for the words that they had rote-learned. This finding needs to be further documented in the future, but we chose to test in a natural school setting rather than to train children with two different methods, because of the ethically questionable global approach, given the knowledge that we have from the behavioral literature. Indeed, teaching with a global method may reinforce the use of non-efficient compensatory strategies already set up by poor readers (e.g., reading by guessing from semantic or visual clues), possibly explaining why non-alphabetical approaches are significantly less efficient in children with learning difficulties (Braibant and Gerard, 1996; Goigoux, 2000; Ehri et al., 2001; Rayner et al., 2001; Deauvieau et al., 2013). Furthermore, if GP mappings cannot be applied, the global method leads to a non-economical storage of written words comparable to visual objects. This in turn could interfere on reading accuracy (e.g., confusion between visually similar words) and reading acquisition, for instance by impeding self-teaching of novel words (Share, 1995), as well as development of phonological awareness and letter knowledge. On the contrary, sufficient GP mappings knowledge allows to infer GP mappings from globally trained words (Byrne, 1984, 1996). Here, good readers could process the global words with an orthography-to-phonology type of process involving the LH, while poor readers tended to rely on whole-word recognition. Therefore, both in poor readers and good readers, our data suggest that the most efficient process “wins the race” (Logan and Cowan, 1984). In good readers, GP mappings are triggered first and efficiently, therefore the LH would be activated automatically whatever the learning method. In poor readers, for whom it is plausible to assume a lack of automatization in GP mappings, visual recognition would be used when possible, similarly to the right hemispheric compensatory mechanisms suggested in dyslexic adults for their deficient left neural network for reading (Kinsbourne et al., 1991; Richlan, 2012; Waldie et al., 2013). Given that visual processes in reading have been suggested to be influenced by gender (Hystegge et al., 2012), we also considered this potential confounding factor in our analyses. Both the distribution of boys/girls in the two subgroups, as well as our supplementary analysis of gender effects indicate that reading ability rather than gender seem to best explain our observation that global words can trigger visual recognition processes.

The phonics and global methods differ by definition on an important aspect, namely item familiarity. Indeed, the phonics method aims at providing the ability to transfer GP mappings on *new* words, while the global method aims at creating representations for the *learnt* items. Therefore, an intrinsic confounded factor inherent to the two methods is that global words were highly familiar to children, while control words and pseudowords were not explicitly taught at school. However, the difference that we observed between groups does not argue in favor of a general familiarity effect, because all children were as familiar to the set of global words. Indeed, we observed that good readers presented a left lateralized response in all conditions: for these children, there was no difference between conditions according to familiarity. In poor readers, on the contrary, the RH engagement for global words could be induced by (visual) familiarity or visual recognition. The inter-conditions correlations by group provide another argument supporting the view that in good readers, the reading processes triggered by all conditions were similar, while for poor readers, they were different. For good readers, the lateralization scores correlated highly between all three conditions (above Rho = 0.75), while for poor readers, correlations between the two control letter strings (words/pseudowords) were significant (Rho = 0.64) but there was no correlation between global words and control letter strings. Finally, the differential processing of global words is also unlikely to be due to a fluctuation of attention given that neither the detection of the color change on the fixation square, nor the overall base rate responses, varied according to conditions and groups.

An open question is how results would be modulated by other language characteristics than French. Indeed, the orthography-phonology consistency plays a crucial role in the efficiency (or not) of applying a GP strategy when learning to read (Ziegler and Goswami, 2005, for a review, Borleffs et al., 2019), or on the sensitivity to novel word structure effects (Lange-Küttner, 2005). More opaque languages like English, or more transparent like Italian, might therefore give rise to different results than observed here. However, the fact that we observed a different lateralization profile for global words only in poor readers shows that the reliance on visual representations for global words is not tied to language *per se*.

In future studies, it would be interesting to assess the persistence of the effects in order to evaluate if the global method, inducing reliance on visual recognition in poor readers, has a negative impact on the long-term development of neural circuits for reading. It may well be that before acquiring the mastery and automatization of GP mappings, “good readers” presented the same neural pattern than “poor readers” for global words. Thus, it is also possible that “poor readers,” after improvement of GP mappings ability with formal instruction, could progressively present a left lateralized response despite the whole-words instruction. Indeed, we have arbitrarily named our groups as “good” and “poor” readers, without assuming any type of disorder in “poor readers.” At this stage of instruction and development, children who knew fewer letters might be only delayed compared to the others, a possibility which was also supported by the weaker performance that they show in all behavioral tests. Thus, it is also possible that “poor readers,” after improvement of GP mappings ability with formal instruction, could progressively present a left lateralized response despite the whole-words instruction. However, we can also assume that if some of these children have a severe or specific disorder and are unable to reach a sufficient level of GP mappings automatization, they could maintain an atypical neural pattern for these global words. Furthermore, as suggested by behavioral studies (Campbell and Butterworth, 1985; Vellutino, 1987), they could even mostly attempt to transfer this strategy to process other written words, in order to compensate their persistent difficulties in acquiring GP mappings.

Our findings lead us to remind education professionals that visual memorization of global word shape is not involved in (adult) expert recognition, which results from the automatization of analytical processes performed on written words. We are aware that the aim of teachers is to vary the approaches in learning to read in order to motivate children, because the process of learning and automatizing all the GP mappings is long and laborious. However, only enhancing GP mappings through alphabetical approaches can provide the indispensable foundations for the development of expert reading skills.

## Data Availability Statement

All datasets generated for this study are included in the article/Supplementary Material.

## Ethics Statement

The studies involving human participants were reviewed and approved by the Biomedical Ethical Committee of the Université catholique de Louvain. Written informed consent to participate in this study was provided by the participants’ legal guardian/next of kin.

## Author Contributions

AL and BR designed the study. AW and AL conducted the study and analyzed the data. AW, AL, BR, and CS wrote the manuscript. BR and CS provided financial support for the researchers and the material.

## Conflict of Interest

The authors declare that the research was conducted in the absence of any commercial or financial relationships that could be construed as a potential conflict of interest.

## Footnotes

1 https://www.letswave.org/

## Supplementary Material

TABLE S1 Complete list of target stimuli used in the FPVS-EEG testing (global words, control words and control pseudowords) in French as well as in their English translation.

TABLE S2 Individual behavioral data of general cognitive functions and reading assessments for the 42 children retained in the study.

DATA SHEET S1 Description of the EEG dataset provided for the 42 children retained in the study (files and data processing steps).

DATA SHEET S2 Compressed folder containing the individual EEG data (frequency domain) for the PF-W condition (control words within pseudofonts).

DATA SHEET S3 Compressed folder containing the individual EEG data (frequency domain) for the PF-PW condition (control pseudowords within pseudofonts).

DATA SHEET S4 Compressed folder containing the individual EEG data (frequency domain) for the PF-GW condition (global words within pseudofonts).

DATA SHEET S5 Analyses of gender effects on both behavioral (general cognitive functions, reading) and FPVS-EEG measures (control words or pseudowords within pseudofonts, global words within pseudofonts; PF-W, PF-PW, PF-GW respectively).

VIDEO S1 22 s excerpt of a stimulation sequence, showing pseudofonts at 6 Hz, with (French) words (PF-W condition) appearing every five items (i.e., 1.2 Hz). The video 1 was generated with a JavaScript (Java SE Version 8) in the MP4 format. During the stimulation, the child is not instructed to read the words but to continuously fixate a central square and press the space bar upon any brief (200 ms) color change of the fixation square (blue to yellow; six changes randomly timed per sequence). This orthogonal task was included to maintain both a central eyes position on the screen and a constant level of attention throughout the entire stimulation.
