# Supplementary material for: Impact of Learning to Read in a Mixed Approach on Neural Tuning to Words in Beginning Readers
Source: Front Psychol. 2020 Jan 23;10:3043. doi: 10.3389/fpsyg.2019.03043 (PMC6989560; doi:10.3389/fpsyg.2019.03043)
Supplement: DATA SHEET S1 — Description of the EEG dataset provided for the 42 children retained in the study (files and data processing steps). [file Data_Sheet_1.pdf]

In total, there are three folders containing the EEG data of the 42 subjects included in the experiment.

There is one folder by condition: "PF-W" (control words inserted in pseudofonts), "PF-PW" (control pseudowords inserted in pseudofonts) and "PF-GW" (global words inserted in pseudofonts).

In each folder, there are two files (.mt and .lw5) for each subject.

Each subject ("S") can be identified under its number and its reading level ("GR" if good reader, "PR" if poor reader, "AR" if average reader).

EEG data analysis were carried out by using Letswave 5.c (a free software ; https://www.letswave.org/) and Matlab 2014 (The Mathworks) according to procedures previously validated (see e.g., Retter & Rossion, 2016).

In each folder, EEG data have been processed in several steps (mentioned under their abbreviations in the files' name) as follows:

1. BDF files (Biosemi 32 channels) importation.

2. Aligning of recordings in a continuous recording when recording has been paused (step "bc").

3. Band-pass filtering between 0.1 and 100 Hz (step "bpf100").

4. Notch filter to remove unshielded electrical noise at 50 Hz (step "notch").

5. Downsampling to 256 Hz in order to reduce file size and data processing time (step "ds_256").

6. Segmentation in order to include 2 s before and after each sequence, resulting in 44 s segments (step "ep_trigger number*_condition name").

7. Linear interpolation in order to replace artifacts or noisy channels (step "Fixed").

8. Re-referencing of all channels to the common average (step "rr").

9. Segmentation from stimulation onset until 39.996 s, corresponding exactly to 48 complete 1.2 Hz cycles within stimulation i.e. largest amount of complete cycles of 833 ms at the deviant stimuli frequency (1.2 Hz) within the 40 s of stimulation period (step "epbin_trigger number").

10. Averaging of the three repetitions of each condition in order to reduce EEG activity that is not phase-locked to the stimulus (step "avg").

11. Fast Fourier Transform applying in order to convert data from the time domain into the frequency domain (step "fft").

SNR transform, baseline subtraction, sum of significant harmonics and grand averages have not been performed.

*Note that the triggers "111", "114" and "122" stands for PF-W, PF-PW and PF-GW condition respectively.
