# Supplementary material for: Impact of Learning to Read in a Mixed Approach on Neural Tuning to Words in Beginning Readers
Source: Front Psychol. 2020 Jan 23;10:3043. doi: 10.3389/fpsyg.2019.03043 (PMC6989560; doi:10.3389/fpsyg.2019.03043)
Supplement: DATA SHEET S5 — Analyses of gender effects on both behavioral (general cognitive functions, reading) and FPVS-EEG measures (control words or pseudowords within pseudofonts, global words within pseudofonts; PF-W, PF-PW, PF-GW respectively). [file Data_Sheet_5.PDF]

ANALYSES OF GENDER EFFECTS

Gender effects have been suggested in reading, with boys relying more on visual processes (Hystegge et al., 2012). To assess whether gender might have an impact of our results, we first tested if the proportion of boys/girls differed as a function of reading level, but this was not the case (*Chi2* (1,37)= 1.303; p=0.254; phi = 0.18). Second, we ran independent t-tests on behavioral measures for the whole sample, comparing boys and girls. None of these comparisons was significant, except for speed in visual attention where boys were slower than girls (see Table here below).

Behavioral tests and sub-tests	Scores: mean (SD)			Independent t-tests	
	Total	Boys	Girls	t-value	p-value
General cognitive functions	N = 42	N = 20	N = 22		
Nonverbal intelligence (CPM, % accuracy)	76.26 (12.20)	76.39 (11.76)	76.14 (12.84)	0.066	0.948
Selective attention (TEA-Ch, speed in sec)	6.75 (2.36)	7.59 (2.23)	5.99 (2.25)	2.315	0.026
Vocabulary production (N-EEL, % accuracy)	77.63 (9.57)	76.22 (10.84)	78.90 (8.29)	-0.904	0.371
Reading ability					
Single letters (BELO, % accuracy)	71.43 (20.48)	70.57 (22.47)	72.20 (18.98)	-0.254	0.801
Composite score (BELO, BALE, % accuracy)	34.57 (18.75)	35.34 (20.18)	33.86 (17.79)	0.254	0.801

Finally, we ran an ANOVA with Hemisphere (LH, RH) x Conditions (PF-PW, PF-W, PF-GW) with a split sample as a function of gender (see histogram below). The results were similar for boys and girls, except that boys were less left-lateralized than girls *in all conditions*, given that this factor did not reach significance in boys (F(1,19)=2.513; p=0.12), while it did in girls (F(1,21)=8.584; p=0.008). Conditions did not differ in any group, boys: (F(2,38)=1.989; p=0.151) , girls (F<1), and the interaction did not reach significance either, boys, (F(2,38)=1.063; p=0.356), girls (F(2,42)=2.151; p=0.129).

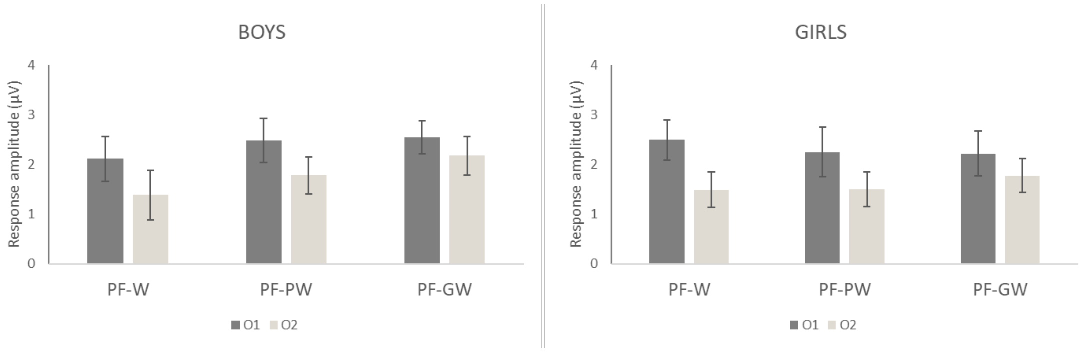

Thus, although visual processes may vary with gender and this may influence reading processes and strategies, in the current sample we do not find any bias in gender distribution for good/poor readers, no differences between boys and girls in behavioral scores of reading, vocabulary, or Raven progressive matrices -which is a visual/perceptual reasoning test. Finally, no difference in the pattern of neural responses across conditions emerged when splitting the file by gender, on the contrary to what happens when we analyzed the impact of reading level.
